# Immunotherapy and the Treatment of Non–Small Cell Lung Cancer

**Published:** 2016-04-01

**Authors:** Suzanne Walker

**Affiliations:** Abramson Center at Penn Presbyterian Medical Center, Philadelphia, Pennsylvania

With more than 200,000 cases expected for 2016, lung cancer is the leading cause of cancer death in both men and women, accounting for more than one-quarter of all cancer deaths.

"A lot of people don’t realize that lung cancer has a higher mortality than breast, prostate, and colorectal cancers combined," said Suzanne Walker, CRNP, MSN, AOCN®, BC, of Abramson Center at Penn Presbyterian Medical Center, Philadelphia, Pennsylvania, "and the 5-year survival is less than 20%. I’m hopeful that with some of our emerging targeted therapies and immunotherapy agents, we’ll definitely see an increase in survival the next time the statistics are reported."

As Ms. Walker reported at the 2015 JADPRO Live conference, held in conjunction with the annual meeting of the Advanced Practitioner Society for Hematology and Oncology (APSHO), more than 50% of cases occur when patients have presented with metastatic disease, and almost one-quarter occur with regional spread. That’s why systemic therapies are so important with this disease, she added. According to Ms. Walker, immunotherapy drugs have become a vital addition to the armamentarium and may well be the future of cancer treatment.

## IMMUNE SYSTEM 101

Immunotherapy, Ms. Walker reviewed, enhances the ability to detect and destroy malignant cells by activating the immune system. "Think of the immune system as providing stimulatory signals," she said, "which would be stepping on the gas pedal or removing those inhibitory signals by lifting off the break."

One prominent pathway for immunotherapy drugs involves programmed cell death protein 1 (PD-1) and programmed cell death ligand 1 (PD-L1), which, Ms. Walker noted, are often mistaken as the same thing. PD-1 is an immunosuppressive molecule that is expressed on many T cells and activated when it binds to its ligand, PD-L1. The activation leads to impaired T-cell function. PD-L1 is expressed on many cancer cells.

T cells, which rely on antigen-presenting cells to "prime" the antigen for recognition, have several mechanisms of action: They can be directly cytotoxic or can recruit or secrete cytokines that can recruit other immune-system cells. And they can also facilitate B-cell activity.

"There are two phases: a priming phase, where the antigen-presenting cell presents to the T cell, and then a connector phase, where the T cell connects with the cancer cell," Ms. Walker explained.

## PEMBROLIZUMAB

Pembrolizumab (Keytruda), a monoclonal antibody that inhibits the action between PD-1 and PD-L1 and PD-L2, is approved by the US Food and Drug Administration (FDA) for patients with metastatic non–small cell lung cancer (NSCLC) whose tumors express PD-L1, as determined by an FDA-approved test. Patients with epidermal growth factor receptor (*EGFR*) or anaplastic lymphoma kinase (*ALK*) genomic tumor aberrations should have disease progression on FDA-approved therapy for these aberrations prior to receiving pembrolizumab.

Much of the data on pembrolizumab in NSCLC, said Ms. Walker, comes from the KEYNOTE-001 trial, a phase IB study of 495 patients with advanced NSCLC. Expression of PD-L1 was assessed using immunohistochemistry. More than 80% of patients had prior therapy. Median overall survival was 12 months, Ms. Walker reported (Garon et al., 2015).

The FDA-approved dosing of pembrolizumab is 2 mg/kg, but in some current trials, researchers are looking at a fixed 200-mg dose. It is a 30-minute infusion administered every 3 weeks.

## NIVOLUMAB

Nivolumab (Opdivo), a human IgG4 monoclonal antibody that binds to the PD-1 receptor and inhibits PD-1 receptor interaction with PD-L1 and PD-L2, is FDA approved for metastatic squamous and nonsquamous NSCLC with disease progression on or after platinum-based chemotherapy. The FDA-approved dosing of nivolumab is 3 mg/kg over 60 minutes every 2 weeks.

"Patients with *EGFR* or *ALK* genomic tumor aberrations should have disease progression on FDA-approved therapy for these aberrations prior to receiving nivolumab," said Ms. Walker. She also noted that, unlike pembrolizumab, with nivolumab, patients do not have to express PD-L1 to be eligible for treatment.

CHECKMATE-017, a phase III study of nivolumab vs. docetaxel in 272 previously treated patients with advanced or metastatic squamous cell NSCLC, showed significant improvement in overall survival (9.2 vs. 6 months favoring nivolumab). Nivolumab also showed a significant reduction in grade 3 or 4 adverse events, from 55% of patients with docetaxel to 7% of patients on nivolumab (Brahmer et al., 2015).

CHECKMATE-057, a phase III randomized trial of nivolumab vs. docetaxel in advanced nonsquamous NSCLC, also showed favorable results, with overall survival increasing from 9.4 months to 12.2 months, favoring nivolumab (Paz-Ares et al., 2015). "These survival rates might not sound huge," stated Ms. Walker, "but patients who respond can have durable responses, and we still haven’t reached the peak of what those responses are."

In the melanoma literature, some patients who have responded to immunotherapy may survive 10 years or more.

"NSCLC literature is much less mature [than the melanoma literature]," she added, "but hopefully we will continue to see those durable responses, which you’re not going to see with chemotherapy."

## PD-L1 STAINING AND PSEUDOPROGRESSION

The role of PD-L1 staining is controversial, according to Ms. Walker. Although some studies have shown improved outcomes for PD-L1–positive patients, others have not.

"This may or may not be predictive and/or prognostic," Ms. Walker reported. "There can also be heterogeneity in the tumor. One sample may be negative, while another is positive. I think the utility is still to be determined. Even patients who are not PD-L1–positive can still respond."

Pseduoprogression is also important to keep in mind when looking at patient response. Imaging studies may demonstrate development of new lesions, inflammatory infiltrates, or an increase in the size of baseline lesions. Immune-related response criteria, Ms. Walker noted, take into account total disease burden, including new measurable lesions, and require confirmatory imaging not less than 4 weeks later.

"We know that patients usually aren’t going to respond for at least 2 months, so it’s important to be careful as to when you’re scanning patients," she said. "There are some new criteria being utilized in some clinical trials to account for pseudoprogression."

## IMMUNE-RELATED SIDE EFFECTS

In addition to the side effects of fatigue and decreased appetite, immunotherapy drugs introduce immune-related side effects, which include pneumonitis, colitis, nephritis/renal dysfunction, endocrine dysfunction, and hepatitis.

"The rates of these immune-mediated side effects are actually very low," said Ms. Walker. "Most are single digits, around the region of 1% to 5%, but it’s still something to be aware of. It’s also important for non-oncology providers to be aware of, so when patients present to the emergency room, the providers will know what to look for and how to manage these symptoms."

## FUTURE DIRECTIONS

"We’re looking at immunotherapy in the adjuvant setting; in oligometastatic disease; in small cell lung cancer; in combination with radiation therapy; and in combination with other immunotherapy and targeted agents, particularly ipilimumab, which works on the CTLA-4 [cytotoxic T-lymphocyte–associated protein 4] area to create a double blockade," Ms. Walker concluded. "This may improve response rates and survival." 
